# Targeted Degradation of MCL-1 by PROTAC Mcl-1 Degrader-1 Exhibits Antiproliferative and Antimigratory Effects and Triggers Mitochondria-Mediated Apoptosis in Colorectal Cancer

**DOI:** 10.3390/cimb48070733

**Published:** 2026-07-17

**Authors:** Seher Saruhan, Deniz Özdemir, Can Ali Ağca

**Affiliations:** 1 Science Institute, Bingöl University, 12000 Bingöl, Türkiye; sehersaruhan33@gmail.com (S.S.); ozdemr.dnz@gmail.com (D.Ö.); 2 Department of Molecular Biology and Genetics, Bingöl University, 12000 Bingöl, Türkiye

**Keywords:** PROTAC, MCL-1, apoptosis, colorectal cancer

## Abstract

Myeloid cell leukemia-1 (MCL-1) is a cellular survival protein belonging to the Bcl-2 protein family and is overexpressed in human colorectal cancer (CRC). Background: This study aimed to reduce cancer progression by suppressing the MCL-1 protein using PROTAC MCL-1 Degrader-1 and Trametinib, with the aim of overcoming the apoptosis resistance caused by high MCL-1 expression levels in colorectal cancer cells. Methods: Therefore, we tested the cell viability, proliferation, mitochondrial membrane potential, cell cycle progression, and cell death potential of MCL1-specific PROTAC Mcl-1 Degrader-1 and the combination of PROTAC Mcl-1 Degrader-1 and Trametinib in colorectal cancer cell lines using different methods such as WST-8 assays, real-time cell analysis, MMP/JC-1 staining assays, flow cytometry, and Western blot analysis. Results: The results suggest that PROTAC Mcl-1 Degrader-1 is associated with dose- and time-dependent reductions in cell proliferation in colorectal cancer cells. Under the experimental conditions used in this study, PROTAC MCL-1 Degrader-1 showed limited effects on the migratory potential of colorectal cancer cells but was associated with changes in cell cycle distribution, including an increased proportion of HT-29 cells in the G2/M phase. In addition, combination treatment with PROTAC MCL-1 Degrader-1 and trametinib was associated with greater reductions in cell viability and proliferation than either monotherapy. The combination treatment was also accompanied by changes in mitochondrial membrane potential and increased apoptotic cell populations, with a higher proportion of early apoptotic cells observed in HT-29 and COLO-205 colorectal cancer cell lines. Conclusion: Our findings suggest that the reduction in MCL-1 protein levels observed following PROTAC Mcl-1 Degrader-1 treatment may contribute to its potential therapeutic relevance in colorectal cancer.

## 1. Introduction

Colorectal cancer, one of the most common cancers worldwide, accounts for almost 9% of cancer-related deaths [1]. Standard treatment options for patients with advanced colorectal cancer include oxaliplatin, irinotecan, and combinations of monoclonal antibodies such as cetuximab, bevacizumab, or panitumumab with fluoropyrimidines. Many patients with advanced colorectal cancer initially respond to combination chemotherapy, but this often results in tumor recurrence and the emergence of drug-resistant tumor cells in later stages [2,3]. The failure of vemurafenib to kill colon cancer cells and the resulting disease recurrence have been reported to be associated with EGFR activation. The addition of EGFR signaling inhibitors has been found to induce synergistic apoptosis by suppressing MEK/ERK activation [4]. Based on these data, it has been shown that apoptosis resistance can be overcome by blocking reactivated MAPK signaling, and in this context, combined treatment with BRAF and MEK inhibition leads to further suppression of MAPK signaling and increased therapeutic efficacy [5]. Apoptosis, a type of programmed cell death under genetic regulation, ensures the safe elimination of cells after they have fulfilled their biological functions [4]. In general, apoptosis resistance is characterized by dysregulated apoptosis signaling, such as an increase in pro-survival proteins (e.g., MCL-1, BCL-2, BCL-XL) and a decrease in pro-apoptotic proteins (e.g., BAX, BAK, BID), which leads to uncontrolled proliferation, tumor survival, and therapeutic resistance [6,7].

MCL-1 has emerged as a critical protein in the BCL-2 family, playing a pivotal role in cell survival, cancer progression, and therapy resistance [6,7]. Overexpression of MCL-1 is associated with resistance to cell death and has been observed in several cancers, including colorectal cancer [8]. This protein affects processes such as the cell cycle, apoptosis, DNA repair, and cellular senescence [9,10], highlighting its importance as a therapeutic target. Scientists have developed various treatment strategies, including small-molecule inhibitors such as AZD5991, which have entered clinical trials (In the phase I clinical trial, patients experienced cardiac complications) for relapsed or refractory leukemia [11,12], though it has not yet been approved by the FDA. Proteolysis-Targeted Chimeras (PROTACs) represent an emerging therapeutic strategy, using bifunctional molecules to degrade specific target proteins rather than merely inhibiting their activity. PROTACs consist of three components: a target protein–ligand, an E3 ligase binder, and a linker [13]. They utilize the ubiquitin–proteasome system to degrade target proteins. Unlike traditional small-molecule inhibitors, which function through the occupancy-oriented model and lose efficacy after dissociating from their target, PROTACs operate via an event-oriented model, continuing to degrade proteins even after dissociation [13,14]. PROTACs offer enhanced specificity and reduced toxicities compared to conventional chemotherapeutics, which often affect normal cells and cause undesirable side effects [15].

Recent advancements have highlighted the potential of PROTACs in targeting MCL-1, which plays a pivotal role in apoptotic resistance. Wang and colleagues developed a PROTAC designed to degrade MCL-1, demonstrating efficacy in the human cervical carcinoma cells (HeLa) [16]. However, despite its demonstrated efficacy in HeLa, the potential role of PROTAC Mcl-1 Degrader-1 has not yet been explored in colorectal cancer. On the other hand, in colorectal cancer cells, the Mek1/2 inhibitor Trametinib has been used to reduce or eliminate the increase in signaling that occurs for the first time. In this study, we aimed to overcome apoptosis resistance in colorectal cancer cells with combination therapy using PROTAC Mcl-1 Degrader-1 and the inhibitor Trametinib. Previous studies have shown that activation of the MEK/ERK pathway contributes to MCL-1 expression and stability. Therefore, concomitant inhibition of MEK signaling with Trametinib and targeting of MCL-1 using PROTAC technology may provide complementary antitumor effects through the disruption of multiple survival pathways. To our knowledge, this is the first study to investigate the effect of PROTAC Mcl-1 Degrader-1 in overcoming apoptotic resistance in colorectal cancer.

## 2. Materials and Methods

### 2.1. Chemicals and Kits

PROTAC Mcl-1 Degrader-1 (compound **C3**) (Catalog number: HY-125877) and Trametinib (Catalog number: HY-10999) were purchased from MedChem Express (Township, NJ, USA) and dissolved in DMSO. The WST-8 assay, used to determine cell viability, was purchased from Elabscience (Houston, TX, USA) (Catalog number: E-CK-A362). The Cell Cycle Analyzer Kit (EcoTech, Nutriculture, Erzurum, Turkey) was used for cell cycle determination (Catalog number: CCA50). The FITC Annexin V Apoptotic Cell Detection Kit was obtained from Bio-Legend (San Diego, CA, USA; Catalog number: 640914). Measurements of mitochondrial membrane potential were completed using JC-1 fluorescent carbocyanine dye obtained from Sigma (St. Louis, MO, USA; CAS number: 47729-63-5).

### 2.2. Cell Lines and Culture Conditions

HT-29 and COLO-205 cells were obtained from ATCC (American Type Culture Col, Manassas, VA, USA). The COLO-205 cell line was cultured in RPMI-1640 medium (St. Louis, MO, USA) containing 10% FBS (Paisley, Scotland, UK). HT-29 was cultured using DMEM medium supplemented with 10% FBS (Paisley, Scotland, UK) and a 1% penicillin/streptomycin (*v*/*v*) mixture.

### 2.3. Cell Viability (WST-8 Test)

Cell viability was analyzed using a colorimetric assay (WST-8). Briefly, 3 × 10^3^ cells per well were seeded into 96-well plates and treated with a combination of PROTAC Mcl-1 Degrader-1 (0.1–0.5–1–2–5–10 µM) at different treatment doses and Trametinib (1 µM) after a 24 h incubation period. At the end of the 48 h treatment period, 20 µL of 5 mg/mL WST-8 staining kit was added to the plates and incubated at 37 °C for 4 h. Absorbance values were read at 450 nm on an ELISA device with a microplate reader (Bio-Rad Benchmark, Hercules, CA, USA). Results were defined as the mean ± standard deviation of three independent experimental results. One-way analysis of variance (ANOVA) followed by Tukey’s multiple comparisons test was used. After obtaining cell viability results, the sigmoidal dose–response equation was used to calculate the IC50 values of PROTAC Mcl-1 Degrader-1 and Trametinib: Y = Low CI + (High CI − Low CI)/{1 + 10^(Log IC50 − X)}. In this equation, Y represents the measured cell index; X is the logarithm of the concentration (M); low CI represents the minimum value; and high CI represents the maximum cell index value [17].

### 2.4. Real-Time Cell Analysis

The effects of the combination of PROTAC Mcl-1 Degrader-1 and Trametinib on cell proliferation in HT-29 and COLO-205 cell lines were determined using the xCELLigence Real-Time Cell Analyzer (ACEA, San Diego, CA, USA). For the experiment, a custom 8-well RTCA-E plate was designed. Each well was filled with 100 µL of complete culture medium for background impedance measurement. Cells (2 × 10^3^) were then seeded, and after seeding, the electronic device in the incubator was placed in 5% CO_2_ and a 24 h incubation period was initiated. After the period, the medium in the well was removed and discarded. Treatments with PROTAC Mcl-1 Degrader-1 (0.1–0.5–1–2–5–10 µM) and Trametinib (1 µM) were administered, and data were recorded throughout the 48 h treatment period. The program was set to record data every 15 min and a CI (cell index) graph was obtained. Data were recorded and analyzed according to the manufacturer’s instructions [18].

### 2.5. Wound Healing-Scratch Assay

Cells (HT-29 and COLO-205) (1 × 10^5^) were seeded into 12-well plates and grown until 100% density was reached. The wound was created as a single layer by scoring it with the tip of a sterile 200 μL pipette (width: ~1 mm). The remaining contaminated cellular debris was washed away with PBS, which was used as serum-free medium. The cells were treated with therapeutic doses of PROTAC Mcl-1 Degrader-1 (0.1–0.5–1–2–5–10 µM) and Trametinib (1 µM) and then allowed to migrate to the bare areas. Images were taken under an inverted microscope at 24 and 48 h intervals. Wound closure was determined using the ImageJ/Wound Healing Size Tool (Version 1.50). The experiment was repeated at least three times. The obtained data are presented as mean ± standard deviation after three independent experiments [19].

### 2.6. Cell Cycle Analysis

Flow cytometry (Beckman Coulter CytoFLEX, Lane Cove West, NSW, Australia; United States of America (USA)) was used to determine the phase of the cell cycle. Propidium iodide is frequently used in cell cycle analysis because its primary purpose is to make cells permeable, bind to double-stranded DNA, and recruit nucleic acids. Cells (HT-29 and COLO-205) (1 × 10^6^ cells/well) were plated in 6-well plates and treated with a combination of PROTAC Mcl-1 Degrader-1 (0.5–1–10 µM) and Trametinib (1 µM) for 48 h after 24 h of incubation. Post-treatment, cells were collected and fixed in cold 70% EtOH for 1 h. The cells were washed with PBS as a washing solution and incubated with 5 μg/mL propidium iodide for 30 min. The results obtained were analyzed using the CytEx-pert program (Version 2.5) to determine the cell percentage. All experiments were independently repeated at least three times [18].

### 2.7. Mitochondria Membrane Potential (ΔΨm) Measurement

JC-1 dye is used to determine membrane potential and accumulates in mitochondria. When it provides a low membrane potential, JC-1 dye exists in a monomeric structure and emits green fluorescence (490 nm excitation/530 nm emission). When it shows a high membrane potential, it enters an aggregation state and produces red fluorescence (530 nm excitation/590 nm emission). A stock solution of 1.5 mM JC-1 dye was prepared in DMSO, used as a chemical solvent. HT-29 and COLO-205 cells (2 × 10^5^) were treated with a combination of PROTAC Mcl-1 Degrader-1 (0.1–0.5–1–2–5–10 µM) and Trametinib (1 µM) for 48 h after 24 h of incubation. The cells were exposed to a 5.0 μM JC-1 staining solution containing PBS for 30 min in a dark environment. The images obtained were acquired using a red and green channel fluorescence microscope (Olympus, Tokyo, Japan). The results, determined as the average red/green fluorescence emission ratio, allow for the quantification of the ΔΨm degree. The results determine the mean ± standard deviation of three independent experiments. The total number and total area were analyzed with ImageJ (Version 1.50i). The color threshold value was used in the area calculation [20].

### 2.8. Annexin V FITC/PI Test

As previously described [18], apoptotic cell death was achieved using flow cytometry with annexin V + staining. After the procedures, the cells were collected into a tube. Cells were washed once with PBS. The washing solution was prepared and suspended in 1× Binding Buffer at a concentration of 1–5 × 10^6^/mL; then 5 µL of fluorochrome-conjugated annexin V was added to 100 µL of cell suspension. Incubation time was 10–15 min at room temperature. HT-29 and COLO-205 cells (1 × 10^6^ cells/well) were washed with 1× Binding Buffer, the suspension was prepared in 200 µL of 1× Binding Buffer, and then 5 µL of Propidium Iodide Staining Solution was added. It was kept in a dark environment at 2–8 °C and analyzed by flow cytometry (Beckman Coulter CytoFLEX) for 4 h. Data were analyzed using the CytExpert program (Version 2.5). All experiments were independently repeated at least three times.

### 2.9. Western Blot

Western blot analysis was performed to determine the effect of PROTAC Mcl-1 De-grader-1 (0.5–1–10 µM) on Mcl-1 and Bcl-2 protein expression in HT-29 and COLO-205 cell lines. The identified proteins (Mcl-1, Bcl-2, Tubulin) were prepared in lysis buffer containing protease inhibitors and added to each sample, followed by incubation at +4 °C for 1 h. Centrifugation was then performed at 14,000 rpm for 10 min at +4 °C. Total protein was determined spectrophotometrically at 595 nm using the Bradford method. Subsequently, equal amounts of total protein were analyzed on SDS-PAGE (10–12%) gels using a Polyacrylamide Gel Electrophoresis (Bio-Rad Trans-Blot cell, Biorad, Hercules, CA, USA) system with 12% gels. Proteins were transferred from the gel to a PVDF membrane and then blocked with 5% nonfat milk powder. They were incubated overnight at 4 °C for treatment with primary antibodies (Mcl-1: 1:500; Bcl-2: 1:500; Tubulin 1:1000). They were then treated with secondary antibodies (anti-mouse IgG-HRP, Jackson, West Grove, PA, USA, 115-035-166, 1:5000 and anti-rabbit IgG-HRP, sc-2357, 1:5000). Species-specific binding was achieved with the secondary antibodies, and images were determined by exposure to X-ray film using the ECL chemiluminescence imaging kit. The intensities of the relevant protein bands were assessed densiometrically using the ImageJ program (Image J; National Institutes of Health, Bethesda, MD, USA). Densitometric readings of the bands were normalized according to tubulin expression. Western blot experiments were performed independently at least three times [21].

### 2.10. Statistical Analysis

All experiments were independently repeated at least three times, and data were presented as mean ± standard deviation (SD). Statistical analyses were performed using GraphPad Prism version 10.0 (GraphPad Software, San Diego, CA, USA). Differences between multiple groups were analyzed using one-way analysis of variance (ANOVA) and Post Hoc Tests, followed by Tukey’s multiple comparison test. A *p*-value < 0.05 was considered statistically significant.

## 3. Results

### 3.1. PROTAC Mcl-1 Degrader-1 and Trametinib Mono and Combination Therapies Suppress Viability of Colorectal Cancer Cells

**Figure 1:**
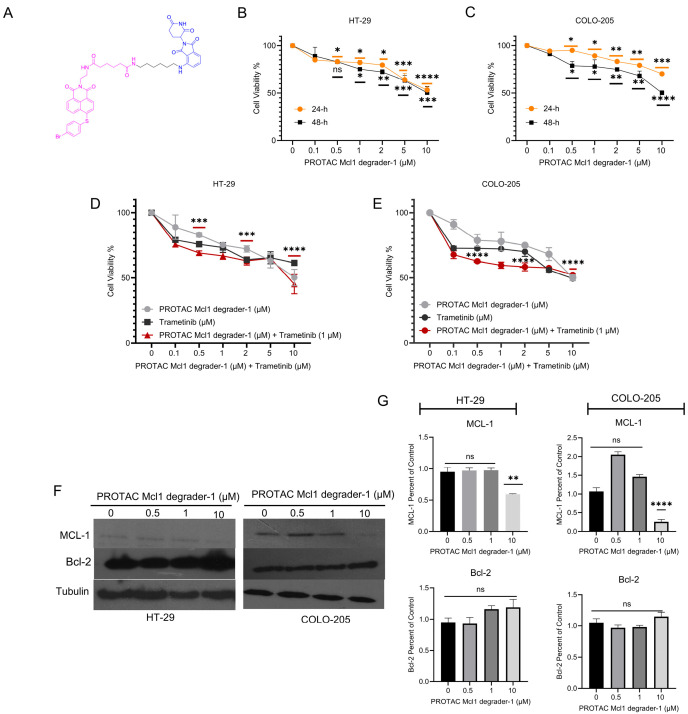
PROTAC Mcl-1 Degrader-1 reduced the viability of HT-29 and COLO-205 cells. (**A**) Molecular structure of PROTAC Mcl-1 Degrader-1 (The pink end of the PROTAC molecule binds to the target protein, while the blue end binds to the E3 ligase). Cell viability of (**B**,**C**) Colorectal cancer cell lines (HT-29/COLO-205) was determined by WST-8 assay after 24 and 48 h of treatment with PROTAC Mcl-1 Degrader-1. (n = 3 replicates). (**D**,**E**) In a 48 h combination therapy where trametinib remained stable at 1 µM and PROTAC Mcl-1 Degrader-1 doses varied, it was shown to effectively reduce the viability of (**D**) HT-29 and (**E**) COLO-205 cells. (**F**) Western blot analysis showed a decrease in MCL-1 protein levels in PROTAC Mcl-1 Degrader-1 colorectal cancer cell lines. Three repetitions were performed in each group. (**G**) Mcl-1 and Bcl-2 protein expressions were statistically evaluated. The results were obtained by one-way ANOVA test * *p* < 0.05, ** *p* < 0.01, *** *p* < 0.001, **** *p* < 0.0001, and ‘ns’ means not significant.

In this study, we first investigated the effect of PROTAC Mcl-1 Degrader-1 on colorectal cancer cell lines using cell viability assays. Cell viability was determined using the WST-8 assay. Under experimental conditions, PROTAC Mcl-1 Degrader-1 reduced cell viability in colorectal cancer cell lines (HT-29 and COLO-205) in a dose- and time-dependent manner (Figure 1B,C). HT-29 (IC50 values for 24 and 48 h: 9.9023 µM, 5.2138 µM) and COLO-205 (IC50 values for 24 and 48 h: 20.6977 µM, 8.9226 µM) cells were susceptible to PROTAC Mcl-1 Degrader-1. Based on the activity of PROTAC Mcl-1 Degrader-1, HT-29 and COLO-205 cell lines were selected for further studies. As a result of the applied combination therapy, it was determined that combination therapy was more effective in reducing cell viability compared to monotherapy. It was determined that the combination of PROTAC Mcl-1 Degrader-1 and the Trametinib inhibitor for 48 h reduced cell viability in the colorectal cancer cell lines HT-29 and COLO-205. Consistent with these results, combination therapy reduced cell viability more effectively than monotherapy (Figure 1D,E). Next, after incubating HT-29 and COLO-205 cells with PROTAC Mcl-1 Degrader-1 for 48 h, we analyzed the effect of PROTAC Mcl-1 Degrader-1 treatment on MCL-1 protein expression using the Western blot method. As seen in the Western blot results, it was statistically demonstrated that at a dose of 10 µM, PROTAC Mcl-1 Degrader-1 reduced MCL-1 protein expression in HT-29 and COLO-205 cell lines, while BCL-2 protein expression remained unchanged (Figure 1F,G).

### 3.2. The Effect of Mono and Combination Therapies of Trametinib with PROTAC Mcl-1 Degrader-1 on Cell Proliferation in Colorectal Cancer Cells

**Figure 2:**
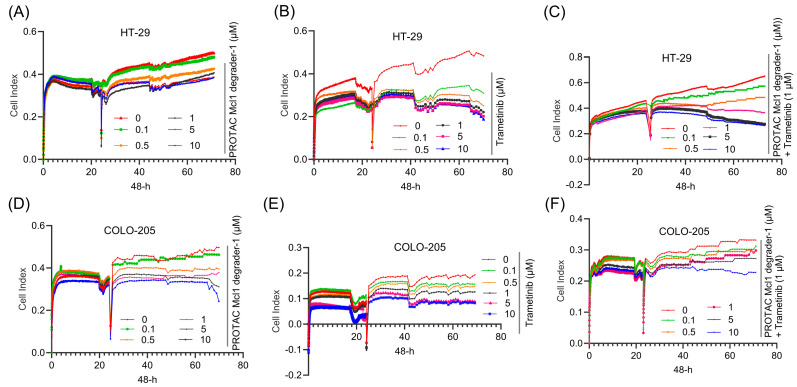
PROTAC Mcl-1 Degrader-1 and Trametinib affect the proliferation of HT-29 and COLO-205 cells. (**A**–**C**) Simultaneous results obtained by Xcelligence Real-Time Proliferation assay after 48 h of treatment of HT-29 cells with (**A**) PROTAC Mcl-1 Degrader-1 and (**B**) Trametinib. Doses are represented by the same colors in the graph. (**D**–**F**) Proliferation results of COLO-205 cell line after 48 h of single and combined treatment with (**D**) PROTAC Mcl-1 Degrader-1 and (**E**) Trametinib, respectively. In combination, a dose of 1 µM Trametinib was used, and treatment was applied with variable doses of PROTAC Mcl-1 Degrader-1.

We evaluated the effect of the combination of PROTAC Mcl-1 Degrader-1 and Trametinib on the proliferation of colon cancer cells using a real-time cell analysis system. HT-29 and COLO-205 cells were treated with PROTAC Mcl-1 Degrader-1 at the indicated concentrations for 48 h. Proliferation in both cell lines decreased in a dose-dependent manner (Figure 2A,D). When the proliferation graph of COLO-205 and HT-29 cell lines was examined after 48 h of treatment with the Trametinib inhibitor, it was observed that cell proliferation decreased in a dose-dependent manner (Figure 2B,E). When the cell proliferation outcome graphs of the combination of PROTAC Mcl-1 Degrader-1 and Trametinib inhibitor in COLO-205 and HT-29 cell lines were examined, it was observed that the treatment doses decreased cell proliferation (Figure 2C,F).

### 3.3. Mono and Combination Therapies with PROTAC Mcl-1 Degrader-1 Inhibit In Vitro Cell Migration in Colorectal Cancer

**Figure 3:**
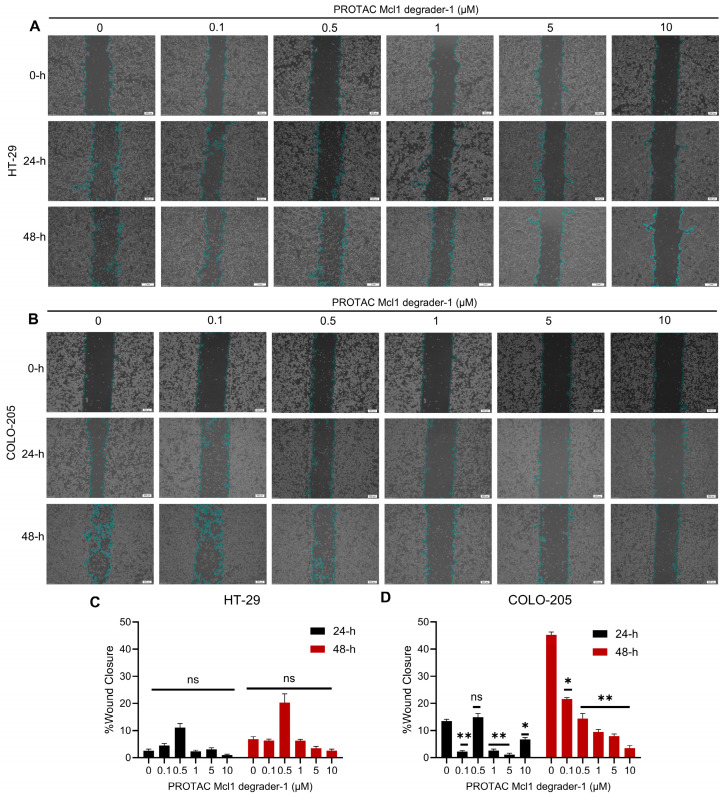
Effect of PROTAC Mcl-1 Degrader-1 on the migratory ability of colorectal cancer cells. (**A**) Wound healing assay was performed under a microscope (original magnification 4×) using an inverted microscope (500 µm scale bar) and images were taken at 0, 24 and 48 time intervals after treatment with different doses of PROTAC Mcl-1 Degrader-1 in HT-29 and (**B**) COLO-205 cell lines. (**C**,**D**) Wound closure analyses of HT-29 and COLO-205 cells were measured using PROTAC Mcl-1 Degrader-1 and microscopic images taken at 24 (black bars) and 48 (red bars) time intervals. Results are presented as mean ± standard deviation (n = 3/group). Statistical significance was determined using one-way ANOVA and Tukey’s multiple comparison test (GraphPad Prism 10.0, GraphPad Software Inc.). * *p* < 0.05; ** *p* < 0.01; ns = not significant.

**Figure 4:**
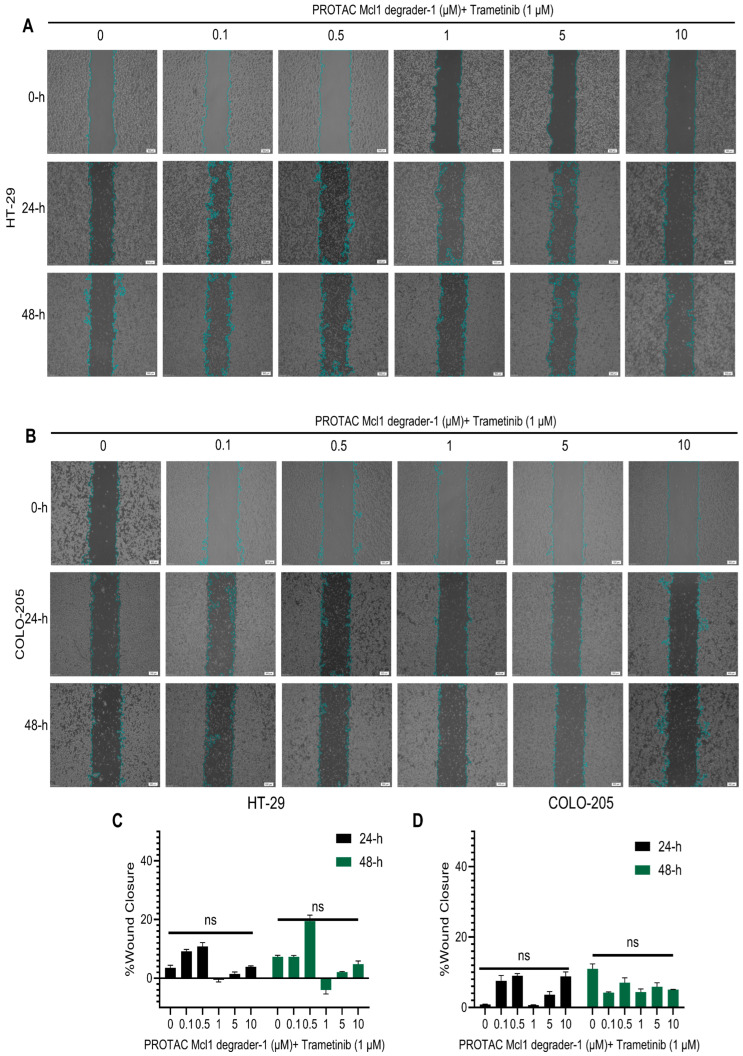
The combination of PROTAC Mcl-1 Degrader-1 and Trametinib did not lead to a statistically significant change in cell migration. (**A**,**B**) Colorectal cancer cells HT-29 and COLO-205 were treated with a combination of PROTAC Mcl-1 Degrader-1 and Trametinib. Trametinib dose was kept constant at 1 µM, while PROTAC Mcl-1 Degrader-1 doses were varied. Cell migration analysis was performed after treatment. Migration capabilities at 0, 24, and 48 h were visualized under a microscope. (**C**,**D**) Graphical analysis of wound closure was performed to determine the migratory ability of cells. The analysis was performed by comparing the 24 and 48 h treatments with the control group (ns = not significant).

In Figure 2, we showed that PROTAC Mcl-1 Degrader-1 did not significantly alter cell migration in the HT-29 cell line but affected the proliferation rate in the COLO-205 cell line. To determine whether PROTAC Mcl-1 Degrader-1 affected the migratory ability of the HT-29 and COLO-205 cell lines, we performed a scratch test. We treated colorectal cancer cell lines with PROTAC Mcl-1 Degrader-1 (0–0.1–0.5–1–5–10 μM) and created scratches. Then, we took images under an inverted microscope at 0–24–48 h intervals (Figure 3A,B). The results of the wound healing test showed no significant difference in wound area closure, but in the COLO-205 cell line, especially at the 48 h treatment, PROTAC Mcl-1 Degrader-1 affected the migratory ability compared to untreated cells (Figure 3C,D). A wound healing test was performed to determine the migratory ability of HT-29 and COLO-205 cell lines after combination therapy with PROTAC Mcl-1 Degrader-1 and a trametinib inhibitor. Microscopic images obtained after the test showed that wound closure did not occur in the HT-29 and COLO-205 cell lines even after 48 h of treatment compared to the control group (Figure 4A,B). As shown in Figure 4C,D, no statistically significant difference was determined.

### 3.4. Effects of PROTAC Mcl-1 Degrader-1 Mono and Combination Therapy on Cell Cycle in HT-29 and COLO-205 Cells

**Figure 5:**
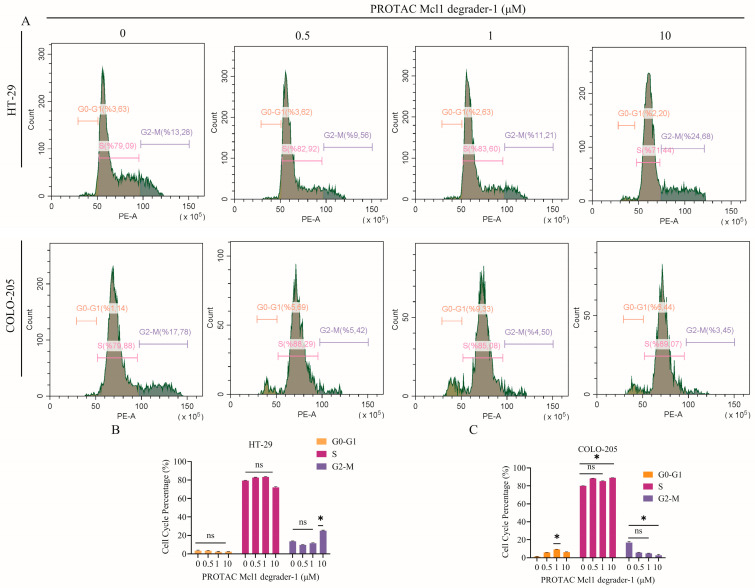
Effect of PROTAC Mcl-1 Degrader-1 on cell count in colorectal cancer. (**A**) Flow cytometric analysis of the preservation of HT-29 and COLO-205 cell counts after 48 h of maintenance with PROTAC Mcl-1 Degrader-1 (n = 3). (**B**,**C**) Statistical analysis of the percentage cell counts of HT-29 and COLO-205 cells. Statistical significance was determined using one-way ANOVA and Tukey’s multiple comparison test (GraphPad Prism 10.0, GraphPad Software Inc.). * *p* < 0.05; ns = not significant.

**Figure 6:**
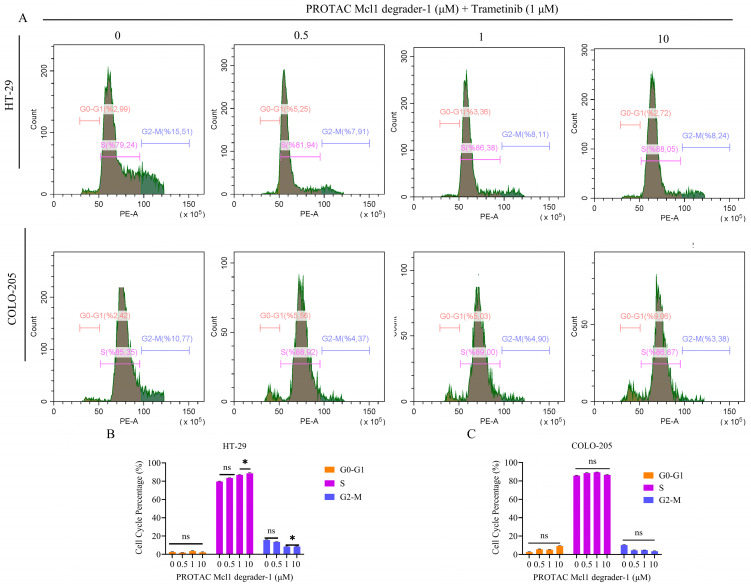
Combination treatment with PROTAC Mcl-1 Degrader-1 and Trametinib for 48 h showed a more pronounced effect on cell cycle distribution in HT-29 cells compared to COLO-205 cells. (**A**) A graph of the cell cycle percentage of HT-29 and COLO-205 cells was plotted after the combination. (**B**,**C**) As shown in the statistical analysis, an effect was observed in the S and G2/M phases in the HT-29 cell line, while no significant effect was statistically demonstrated in the COLO-205 cell line. Statistical significance was determined using one-way ANOVA and Tukey’s multiple comparison test (GraphPad Prism 10.0, GraphPad Software Inc.). * *p* < 0.05; ns = not significant.

To investigate the effect of PROTAC Mcl-1 Degrader-1 on cell cycle arrest, we examined cell cycle patterns after treatment. We treated HT-29 and COLO-205 cell lines with PROTAC Mcl-1 Degrader-1 (0–0.5–1–10 μM) for 48 h. After treatment, we fixed them in cold 70% EtOH for 1 h. Then, we incubated the cells with 5 μg/mL propidium iodide for 30 min. Finally, we determined the percentage of cells in the cell cycle phase with the CytExpert program (Version 2.5). The results showed that exposure of HT-29 cells to PROTAC Mcl-1 Degrader-1 was associated with an increase in the G2/M phase cell ratio, while a slight increase in the G0/G1 population was observed in COLO-205 cells (Figure 5A,C). Flow cytometry plots used to assess the cell cycle were examined in HT-29 and COLO-205 cell lines after combination therapy with PROTAC Mcl-1 Degrader-1 and the inhibitor Trametinib. In HT-29 cells, an increase in the ratio of cells in the S and G2/M phases was observed compared to the control group, while no significant change was detected in COLO-205 cells (Figure 6A). In addition, combination therapy was found to be associated with an increase in the ratio of cells in the S and G2/M phases and a simultaneous decrease in the G0/G1 phase in HT-29 cells; this suggests a possible dose-dependent trend under the experimental conditions used in this study (Figure 6B,C).

### 3.5. PROTAC Mcl-1 Degrader-1 and Trametinib Mono and Combination Disrupt Mitochondrial Membrane Integrity in HT-29 and COLO-205 Cells

**Figure 7:**
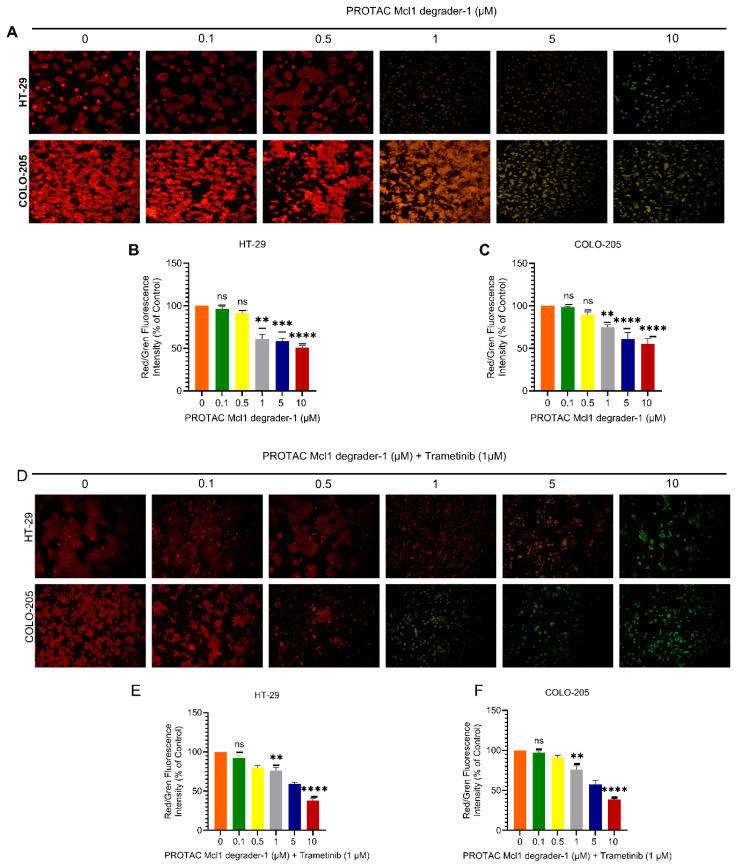
The combination of PROTAC Mcl-1 Degrader-1 and Trametinib affected mitochondrial membrane potential. (**A**) HT-29 and COLO-205 cell lines were stained with JC-1 after treatment with PROTAC Mcl-1 Degrader-1 for 48 h and imaged under a fluorescence microscope (original magnification 20×). The yellow-orange fluorescence of JC-1 dimers was found in cell regions with high mitochondrial membrane potential, while the green fluorescence of JC-monomers was prevalent in cell regions with low mitochondrial membrane potential. (**B**,**C**) Percentage red/green fluorescence intensity plots showed that HT-29 and COLO-205 cells were converted to MMP in both cell lines after 48 h of treatment with PROTAC Mcl-1 Degrader-1, particularly at the final dose of 10 μM. (**D**) Cell lines treated with a combination of PROTAC Mcl-1 Degrader-1 and Trametinib at different 48 h doses were subjected to JC-1 staining. After staining (original magnification 20×), images were acquired under a fluorescence microscope. (**E**,**F**) Quantitative analysis of the transition from mitochondrial orange to green coloration between different combination treatment groups. The statistical value of this experiment was determined using one-way ANOVA and Tukey’s multiple comparison test (GraphPad Prism 10.0, GraphPad Software Inc.). ** *p* < 0.01; *** *p* < 0.001; **** *p* < 0.0001; ns = not significant.

Here, we evaluated the effect of PROTAC Mcl-1 Degrader-1 on mitochondrial membrane potential (ΔΨm) in colorectal cancer cells (Figure 7A–C). We measured ΔΨm using JC-1 to assess mitochondrial membrane potential and integrity. Treatment with PROTAC Mcl-1 Degrader-1 resulted in a decrease in ΔΨm, while changes in cell morphology were observed in both cell lines (Figure 7C). Our results demonstrated that PROTAC Mcl-1 Degrader-1 affects mitochondrial membrane integrity in HT-29 and COLO-205 cells (Figure 7B,C). Microscopic images were recorded from HT-29 and COLO-205 cell lines after combined treatment with the PROTAC Mcl-1 Degrader-1 inhibitor and Trametinib. At doses of 0.1 and 0.5 µM, no statistically significant effect on membrane potential was observed compared to the control group. However, increasing the dose led to changes in cell morphology, a decrease in cell number, and a change in color to green (Figure 7D). As seen in the fluorescence intensity graph, a decrease in mitochondrial membrane integrity was observed, especially at the highest dose.

### 3.6. Effects of Mono and Combination Therapy of Trametinib with PROTAC Mcl-1 Degrader-1 on Programmed Cell Death Apoptosis in HT-29 and COLO-205 Cells

**Figure 8:**
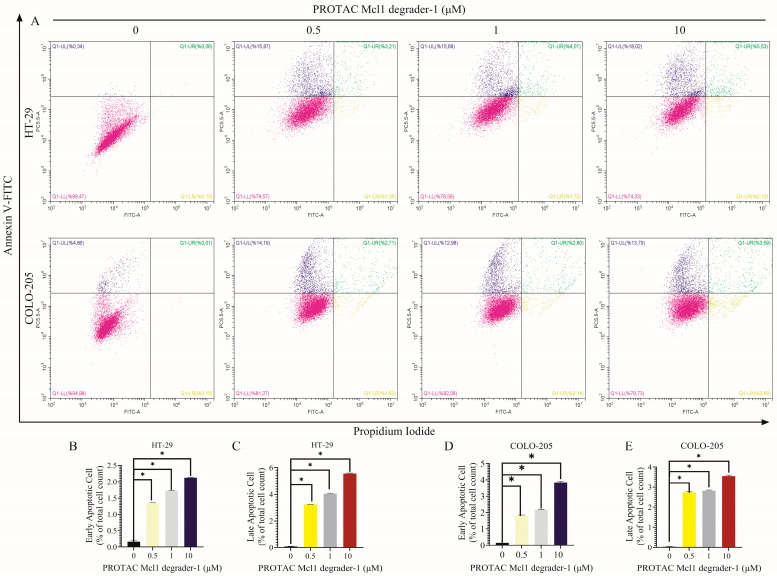
PROTAC Mcl-1 Degrader-1 alone was associated with a partial increase in apoptotic cell populations. (Q1 LL (Control), Q1 UL (Necrosis), Q1 UR (Late apoptosis), Q1 LR (Early apoptosis). (**A**) After 48 h of PROTAC Mcl-1 Degrader-1 treatment of HT-29 and COLO-205 cells, Annexin V and PI-labeled cell apoptosis was analyzed by flow cytometry. (**B**,**C**) Statistical analysis of cells in (**B**) early and (**C**) late apoptosis phases of HT-29 cell line after treatment with PROTAC Mcl-1 Degrader-1. (**D**,**E**) Statistical analysis of COLO-205 cells in (**D**) early and (**E**) late apoptosis phases after treatment with PROTAC Mcl-1 Degrader-1. The obtained results were presented using mean ± standard deviation (n = 3/group). Statistical significance in the experiment was determined using one-way ANOVA and Tukey’s test for multiple comparisons (GraphPad Prism 10.0, GraphPad Software Inc.). * *p* < 0.05.

**Figure 9:**
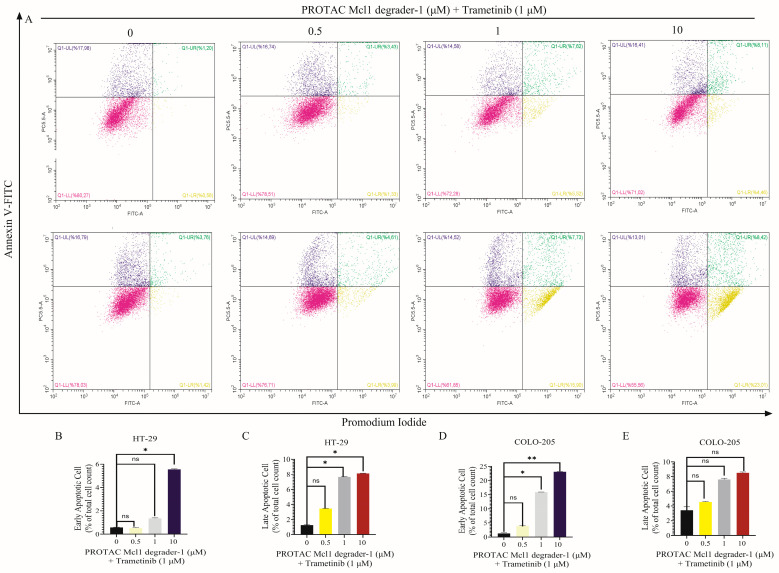
The combination of PROTAC Mcl-1 Degrader-1 and trametinib was observed to be associated with an increase in apoptotic cell populations. (Q1 LL (Control), Q1 UL (Necrosis), Q1 UR (Late apoptosis), Q1 LR (Early apoptosis). (**A**) The degree of apoptosis in HT-29 and COLO-205 cells was monitored by flow cytometry after 48 h of exposure to a combination of PROTAC Mcl-1 Degrader-1 and Trametinib. Combination therapy was shown to be associated with a higher level of apoptosis compared to monotherapy. (**B**,**C**) Treatment of the HT-29 cell line with the combination of PROTAC Mcl-1 Degrader-1 and Trametinib showed increased early and late apoptosis graphs, indicating the stages of apoptosis, compared to the control group. (**D**,**E**) The outcome of combination therapy in the COLO-205 cell line shows a significant effect on early apoptosis in the early and late apoptosis graphs. Statistical analysis after combination therapy showed that the final dose of 1 µm Trametinib and 10 µm PROTAC Mcl-1 Degrader-1 increased early apoptosis by 23% and late apotosis by 8%, particularly in the COLO-205 cell line. The values are expressed as the mean ± SD (n = 3/group). Statistical significance was determined using one-way ANOVA followed by Tukey’s multiple comparison test. * *p* < 0.05; ** *p* < 0.01; ns = not significant.

We analyzed whether PROTAC Mcl-1 Degrader-1 triggered programmed cell death using flow cytometry. First, we treated HT-29 and COLO-205 cell lines with PROTAC Mcl-1 Degrader-1 (0–0.5–1–10 μM) for 48 h (Figure 8A). After treatment, we added Binding Buffer and suspended the cells. We analyzed Annexin V staining and propidium iodide uptake by flow cytometry. Compared to the control group, a dose-dependent increase in early and late apoptotic cell populations was observed in cells treated with PROTAC Mcl-1 Degrader-1 (Figure 8B–E). Overall, these findings indicate that PROTAC Mcl-1 Degrader-1 is associated with an increase in apoptotic cell death in HT-29 and COLO-205 cells under the experimental conditions used in this study. Following combination therapy with PROTAC Mcl-1 Degrader-1 and trametinib, Annexin V analysis showed a dose-dependent increase in apoptotic cell populations in both HT-29 and COLO-205 cell lines compared to the control group (Figure 9A–E). This trend appears to be more pronounced at higher concentrations.

## 4. Discussion

Mcl-1, a key survival protein of the Bcl-2 family, is an essential regulator of tumor maintenance and resistance to therapy in multiple cancer types [6,10]. Its high expression in colorectal cancer potentiates oncogenic MAPK/ERK signaling and promotes apoptosis resistance [22,23]. While several Mcl-1 antagonists, such as AZD5991, have progressed into clinical testing [11,12], on-target toxicities, most notably cardiotoxicity-mediated clinical hold of AMG-397 (NCT03465540), have limited the translation potential of these molecules [22]. These shortcomings highlight the challenge for alternative approaches. PROTAC Mcl-1 Degrader-1 degradation provides a mechanistically distinct route with superior selectivity and likelihood of evading toxicity-related hurdles [24]. Colorectal tumors are reliant on high levels of Mcl-1 expression, and therefore Mcl-1 degradation in these tumors is an attractive precision-therapeutic option [25]. The application of PROTAC, designed to induce the degradation of the MCL-1 protein, opens up innovative avenues for therapeutic intervention, particularly in apoptotic resistance, where MCL-1 plays a pivotal role. Wang and colleagues developed a PROTAC that pharmacologically targets degrading MCL-1 and demonstrated its efficacy in HeLa cell lines [16]. Despite PROTAC Mcl-1 Degrader-1 demonstrating efficacy in human HeLa cell lines, its potential role in colorectal cancer remains unexplored. Therefore, for the first time, we investigated the effect of PROTAC Mcl-1 Degrader-1 in human colorectal cancer. Moreover, other PROTACs can target Bcl-2 and Mdm2 proteins in addition to Mcl-1. However, PROTAC Mcl-1 Degrader-1 only targets Mcl-1 and causes its degradation. Although PROTAC Mcl-1 Degrader-1 has not yet received regulatory approval, it was selected for this study based on its reported ability to target MCL-1, a key anti-apoptotic protein that plays a critical role in colorectal cancer cell survival and resistance to apoptosis. Given that MCL-1 is considered an important therapeutic target in colorectal cancer, the present study was designed as a proof-of-concept approach to investigate whether the simultaneous targeting of MCL-1 and the MEK/ERK signaling pathway through combination treatment with Trametinib could enhance antitumor activity and provide potential therapeutic benefits.

The effect of PROTAC Mcl-1 Degrader-1 on cell viability and proliferation was evaluated using cell culture experiments on colorectal cancer cells. The results showed that PROTAC Mcl-1 Degrader-1 (<10 µM) reduced the viability of HT-29 and COLO-205 cells. The results revealed that PROTAC Mcl-1 Degrader-1 reduced the viability of colorectal cancer cells. A real-time cell proliferation assay was used to determine whether a treatment could inhibit cancer cell growth or trigger cell death via apoptosis. The findings showed that PROTAC Mcl-1 Degrader-1 reduced the proliferation of HT-29 and COLO-205 cells. Cell viability and proliferation analyses were performed on colorectal cancer cell lines HT-29 and COLO-205 48 h after administration of combination therapy with PROTAC Mcl-1 Degrader-1 and Trametinib. Graph analysis showed that PROTAC Mcl-1 Degrader-1 and Trametinib alone reduced cell viability in both HT-29 and COLO-205 cell lines, but the reduction was more effective with combination therapy compared to monotherapy. Cell proliferation assays showed a significant reduction in cell proliferation in both cell lines compared to monotherapy. Although the combination treatment demonstrated significant antitumor activity in vitro, the effective concentrations used in this study may be relatively high from a translational standpoint. Consequently, these findings should be interpreted as proof-of-concept evidence of biological activity. Additional pharmacokinetic, pharmacodynamic, and in vivo studies will be necessary to establish the clinical relevance and therapeutic feasibility of this approach. Doi et al., who obtained similar viability results to our study, used Maritoclax and ABT737 to degrade Mcl-1. Their study results showed that combination therapy with inhibitors reduced cell viability more effectively than monotherapy [26]. Many studies have shown that MCL-1 plays a role in epithelial–mesenchymal transition (EMT), which regulates invasiveness and migration [27,28,29]. Therefore, we investigated the efficacy of PROTAC Mcl-1 Degrader-1 on the migratory ability of colorectal cancer cell lines using a wound healing assay. A cell line-dependent effect on wound healing was observed after PROTAC Mcl-1 Degrader-1 treatment; a significant effect was seen in COLO-205 cells, while no noticeable change was detected in HT-29 cells, even at 10 µM. Overall, these findings suggest that under the applied experimental conditions, PROTAC Mcl-1 Degrader-1 may have limited effects on cell migration in the colorectal cancer cell lines used in this study. MCL-1 has been implicated in the regulation of various phases of the cell cycle, and genetic deletion or pharmacologic inhibition of MCL-1 consistently connects MCL-1 perturbation to the cell cycle [10]. Previous studies have shown that MCL-1 interacts with cyclin-dependent kinases (CDKs), which play central roles in cell cycle checkpoints and progression [30]. Several studies have shown that MCL-1 inhibition leads to both cell cycle arrest in the G1 phase [31] and also in the G2/M phase [32]. To investigate the potential effect of PROTAC Mcl-1 Degrader-1 on the cell cycle, an increase in the G2/M phase was observed in both cell lines treated with PROTAC Mcl-1 Degrader-1; this indicates a change in cell cycle progression in the HT-29 cell line compared to untreated cells. Notably, an increase in the G0-G1 phase was observed in PROTAC Mcl-1 Degrader-1-treated COLO-205 cells. Flow cytometry graphs of HT-29 and COLO-205 cell lines were examined to determine the cell cycle after combination therapy with the PROTAC Mcl-1 Degrader-1 inhibitor and the Trametinib inhibitor. In HT-29, a dose-dependent increase in the S phase and G2-M phase and a decrease in the G0 phase were observed in both treatment groups. In contrast, no significant changes were observed in the COLO-205 cell line. Overall, these findings suggest that PROTAC Mcl-1 Degrader-1 may be associated with alterations in cell cycle distribution in HT-29 cells, while showing limited effects in COLO-205 cells under the experimental conditions used in this study. MCL-1 is involved in not only cell cycle regulation but also mitochondrial function [33].

CL-1 plays a crucial role in stabilizing mitochondrial membrane potential (MMP) and contributing to the inhibition of apoptosis in cancer cells [34,35]. Given the critical role of MCL-1 in the induction of apoptosis and mitochondrial membrane potential, we investigated the effect of PROTAC Mcl-1 Degrader-1 on MMP using the JC-1 fluorescence (ΔΨm) dye to determine the PROTAC Mcl-1 Degrader-1-mediated effect of MMP loss. In this study, a decrease in red fluorescence intensity (JC-1, ΔΨm) was observed in HT-29 and COLO-205 cells treated with PROTAC Mcl-1 Degrader-1 compared with the untreated group. These findings suggest that PROTAC Mcl-1 Degrader-1 may be associated with changes in mitochondrial membrane potential under the experimental conditions used in this study. Several previous studies have identified the role of Mcl-1 in avoiding apoptosis and its anti-apoptotic activity by inhibiting pro-apoptotic proteins involved in the intrinsic apoptosis pathway, a mitochondria-mediated pathway.

The MCL-1 inhibitor AZD5991, currently under clinical investigation, has been shown to induce apoptosis in MCL-1-dependent cancer cells [17]. Another MCl-1 inhibitor (S63845) used in a preclinical study also induced programmed cell death by inducing BAX/BAK-dependent apoptosis [11]. The flow cytometry results showed that PROTAC Mcl-1 Degrader-1 induced both early and late apoptosis at doses of less than 10 µM. Consistent with previous studies, PROTAC Mcl-1 Degrader-1 treatment was associated with alterations in MCL-1 protein levels in HT-29 and COLO-205 cells, along with an increase in both early and late apoptotic cell populations. In HT-29 and COLO-205 cells, Annexin V analysis indicated an increase in apoptotic cell populations following combination treatment with PROTAC Mcl-1 Degrader-1 and trametinib compared with monotherapy and control groups. This increase appeared to be more pronounced with increasing concentrations. Overall, combination treatment was associated with higher apoptotic cell levels compared with single-agent treatments in both cell lines under the experimental conditions used in this study. Furthermore, the study by Lee and colleagues aimed to demonstrate that suppressing Mcl-1 signaling in colorectal cancer cell lines leads to TRAIL-induced apoptosis. The Annexin V test results showed a significantly higher rate of delayed apoptosis in cells treated with combination therapy compared to monotherapy [35].

### Strengths and Limitations of the Present Study

One of the strengths of this study is that it is the first to comprehensively evaluate the monotherapy of PROTAC Mcl-1 Degrader-1 and its combination therapy with trametinib on the colorectal cancer cell lines HT-29 and COLO-205. Furthermore, instead of focusing on a single outcome, this study simultaneously assessed multiple endpoints, including cell viability, proliferation, cell migration, cell cycle, mitochondrial membrane potential, apoptosis, and the expression levels of MCL-1/BCL-2 proteins via Western blot analysis. The evaluation of numerous complementary biological assays allowed for a better understanding of the cellular responses to therapies and enabled a more detailed assessment of the potential effects of combination therapy. As a result of these findings, our study provides additional evidence for the growing research into MCL-1-targeted therapeutic approaches for colorectal cancer treatment and offers a useful foundation for future mechanistic and preclinical studies.

Despite the strengths of our study, several limitations must be considered. First, all experiments used colorectal cancer cell lines HT-29 and COLO-205, and these experiments were performed only in vitro; therefore, the findings should be interpreted cautiously until they are validated under appropriate in vitro conditions. Secondly, although changes in MCL-1 expression were observed following treatment with PROTAC Mcl-1 Degrader-1, mechanistic analyses such as proteasome inhibition, ubiquitination assays, or protein half-life assays, which are necessary to directly address the degradation of PROTAC Mcl-1 Degrader-1, were missing. Furthermore, Western blot analysis only evaluated MCL-1 and BCL-2 protein expression; additional signaling molecules involved in apoptosis and cell cycle regulation were not investigated. Another limitation of this study is that the biological effects of PROTAC Mcl-1 Degrader-1 were not tested in a healthy human colon epithelial cell line. The fact that it has only been evaluated in colorectal cancer cell lines (HT-29 and COLO-205) limits the assessment of the selectivity and potential toxicity of the treatment towards non-malignant cells. Therefore, future studies involving different colorectal cancer cell lines and healthy colon epithelial cells will be important to further validate the biological effects and therapeutic potential of this treatment strategy. Consequently, a complete elucidation of the molecular mechanisms underlying the obtained biological responses is necessary. Studies including comprehensive mechanistic analyses and in vivo validation are needed to confirm the current findings and further determine the therapeutic potential of PROTAC Mcl-1 Degrader-1, both alone and in combination with trametinib, in colorectal cancer.

Future studies should focus on elucidating in more detail the molecular mechanisms underlying the biological effects of PROTAC Mcl-1 Degrader-1. In particular, mechanistic studies including proteasome inhibition, ubiquitination assays, and protein half-life analyses will provide more accurate evidence about the mechanism of action of PROTAC Mcl-1 Degrader-1. In addition, assessing the expression and activation of key regulators (including caspases) involved in cell cycle progression (such as cyclins) and apoptosis will contribute to a more comprehensive understanding of the cellular responses to be achieved. Furthermore, it seems necessary to confirm the present findings in appropriate in vivo models to evaluate the therapeutic potential and safety profile of PROTAC Mcl-1 Degrader-1. Finally, extension of these studies to additional colorectal cancer cell lines and patient-derived models with diverse molecular characteristics will help determine the broader applicability and translational significance of the findings.

## 5. Conclusions

Overall, the results obtained in this study demonstrate that PROTAC Mcl-1 Degrader-1 reduces MCL-1 protein levels and proliferation. PROTAC Mcl-1 Degrader-1 treatment has been associated with changes in cell cycle distribution and increased apoptotic cell death in colorectal cancer cells. These effects appear to occur in conjunction with changes in mitochondrial membrane integrity under the experimental conditions used in this study. The findings demonstrate that combination therapy with the PROTAC Mcl-1 Degrader-1 inhibitor and Trametinib has a significant effect compared to monotherapy treatments in colorectal cancer cell lines. Although PROTAC Mcl-1 Degrader-1 treatment shows promising results in colorectal cancer cells, further studies are needed to address the underlying molecular mechanisms and establish a direct causal relationship between MCL-1 degradation and the observed apoptotic response. Our findings suggest a novel avenue for colorectal cancer treatment.

## Data Availability

The original contributions presented in this study are included in the article. Further inquiries can be directed to the corresponding author.

## Abbreviations

The following abbreviations are used in this manuscript:

ANOVA	Analysis of Variance
BCL-2	Hücreli Lenfoma
CCK-8	Cell Count Kit-8
CDKs	Cyclin-Dependent Kinases
CRC	Colorectal Cancer
DCFDA	Dichlorodihydro Flurescein Diacetate
DMEM	Dulbecco’s Modified Eagle’s Medium
dsDNA	Double-stranded Deoxyribonucleic Acid
EMT	Epithelial–Mesenchymal Transition
FBS	Fetal Bovine Serum
IC50	Half Maximal Inhibitory Concentration
JC-1	5,5′,6,6′-Tetrachloro-1,1′,3,3′-tetraethylbenzimidazolylcarbocyanine, iodide
MCL-1	Myeloid Cell Leukemia
MMP	Mitochondrial Membrane Potential
PROTAC	Proteolysis Targeted Chimera
PVDF	Polyvinylidene Fluoride
ROS	Reactive Oxygen Species
RPMI-1640	Roswell Park Memorial Institute-1640
ΔΨm	Mitochondrial Membrane Potential
